# Alternative antibiotic regimens improve palatability and welfare in mice for gut bacterial depletion

**DOI:** 10.1038/s41684-026-01728-3

**Published:** 2026-05-11

**Authors:** Kaoutar Abaakil, Yang Bi, Laetitia Firmenich, Xuan Yang, Mariam Alireza, Shahriar Mueed, Monica Campos, Marko Storch, Despoina Chrysostomou, Julian R. Marchesi, Jia V. Li

**Affiliations:** 1 Division of Digestive Diseases, Department of Metabolism, Digestion and Reproduction, Faculty of Medicine, Imperial College London, London, UK; 2 London Biofoundry, Department of Infectious Disease, Faculty of Medicine, Imperial College London, London, UK

**Keywords:** Microbiome, Metabolomics, Mouse

## Abstract

Here we evaluated vancomycin–neomycin or enrofloxacin–ampicillin treatments as alternatives to the poorly tolerated ampicillin–metronidazole–vancomycin–neomycin cocktail for gut bacterial depletion in mice. Both regimens were well tolerated in drinking water and did not reduce food intake or body weight. Metabolic profiling showed reduced bacterial metabolic activity in both groups, but a marked reduction in total bacterial abundance only in the enrofloxacin–ampicillin group. These findings support enrofloxacin–ampicillin as a refined antibiotic strategy for gut bacterial depletion, improving palatability and welfare.

## Abstract (web summary)

The authors evaluated two alternative antibiotic regimens to refine gut bacterial depletion in mice. Although both regimens were better tolerated than the standard cocktail, only enrofloxacin–ampicillin reduced bacterial load.

## Main

**Fig. 1:**
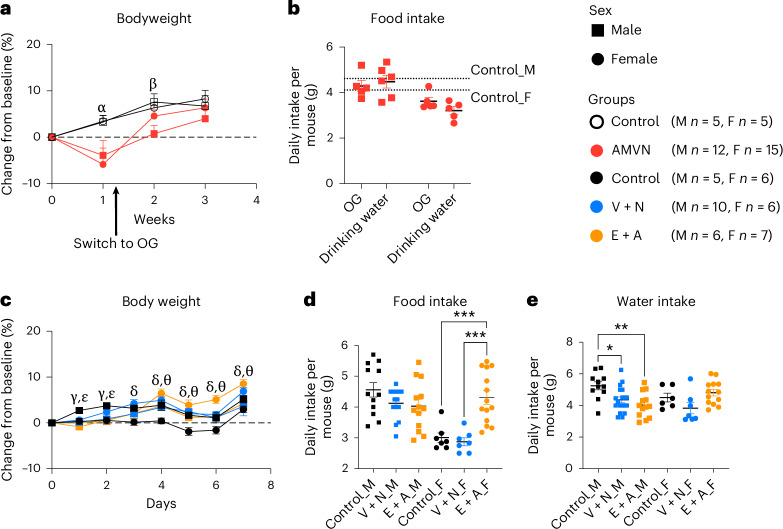
Effects of antibiotic regimens on body weight, food intake and water intake. **a**, Body weight changes in Fabpl*Cre*;*Apc*^15lox/+^ mice treated with AMVN cocktail and controls. α and β denote significant differences between AMVN and controls in females (F) and males (M), respectively (mixed-effects model with Benjamini–Hochberg correction, *P* < 0.05). **b**, Average daily food intake per AMVN-treated mouse during AMVN administration through drinking water and OG, calculated from weekly food consumption measured per cage. No significant difference was detected (one-way analysis of variance (ANOVA) with Šidák’s multiple comparisons test). **c**, Body weight changes in mice treated with vancomycin and neomycin (V + N), enrofloxacin and ampicillin (E + A) or control water. γ and ε denote significant differences in V + N- or E + A-treated male mice compared with control, and δ and θ denote these significances in females (two-way ANOVA with Benjamini–Hochberg correction, *P* < 0.05). **d**,**e**, Food (**d**) and water (**e**) intake of V + N, E + A and control groups measured daily per cage over 1 week (one-way ANOVA with Šidák’s multiple comparisons test, *P < 0.05, **P < 0.01, ***P < 0.001). All data are presented as mean ± standard error of mean.

Antibiotics have been widely used in mouse models to deplete the gut bacterial abundances for studying gut microbiome function. A commonly used antibiotic cocktail, consisting of ampicillin, metronidazole, vancomycin and neomycin (AMVN), is typically administered via drinking water^1,2^. However, we (Fig. 1a) and others^3,4^ found that this method can substantially reduce water palatability, leading to dehydration and critical weight loss in mice. This effect could be attributed to the metallic-like odor of metronidazole. Sugars as palatability enhancers have been used to improve water consumption^5^, but their use could confound scientific findings as sugars can alter bacterial metabolism and composition^6^. An alternative method for antibiotic administration is through oral gavage (OG)^4^, which in our study proved successful, as mouse body weights recovered within a week (Fig. 1a). However, OG and handling stress can induce physiological changes that impact research findings^7,8^. Furthermore, OG carries risks of procedural complications, for example, accidental damage to the esophagus or stomach, and accidental tracheal administration^9,10^. Notably, food intake did not differ significantly between AMVN administration via drinking water or OG (Fig. 1b), highlighting that weight loss was primarily driven by reduced water intake.

Given these limitations, we tested two alternative regimens: vancomycin and neomycin (V + N), and enrofloxacin and ampicillin (E + A)^3^, administered through drinking water. Vancomycin and neomycin primarily target Gram-positive^11^ and Gram-negative^12^ bacteria, respectively, whereas enrofloxacin and ampicillin are broad-spectrum antibiotics^13,14^; these combinations aimed to deplete a wide range of gut bacteria. We evaluated the effects of these antibiotic combinations on body weight, water and food intake, urinary and fecal metabolome and fecal bacterial compositions of Fabpl*Cre*;*Apc*^15lox/+^ mice, a genetic model for colorectal cancer^15^.

We included three experimental groups: non-antibiotic-treated control (male *n* = 5; female *n* = 6), V + N (male *n* = 10; female *n* = 6) and E + A (male *n* = 6; female *n* = 7) treatments. Body weights and food intake of males were comparable between antibiotics-treated and control groups (Fig. 1c,d). By contrast, antibiotics-treated females gained weight over time (Fig. 1c) and food intake of E + A-treated females was significantly higher than that of the control group (Fig. 1d). E + A and V + N lowered the water consumption of male mice compared with the control, but no differences were observed in female mice (Fig. 1e). Despite these variations, mice seemed active and maintained stable body weights.

**Fig. 2:**
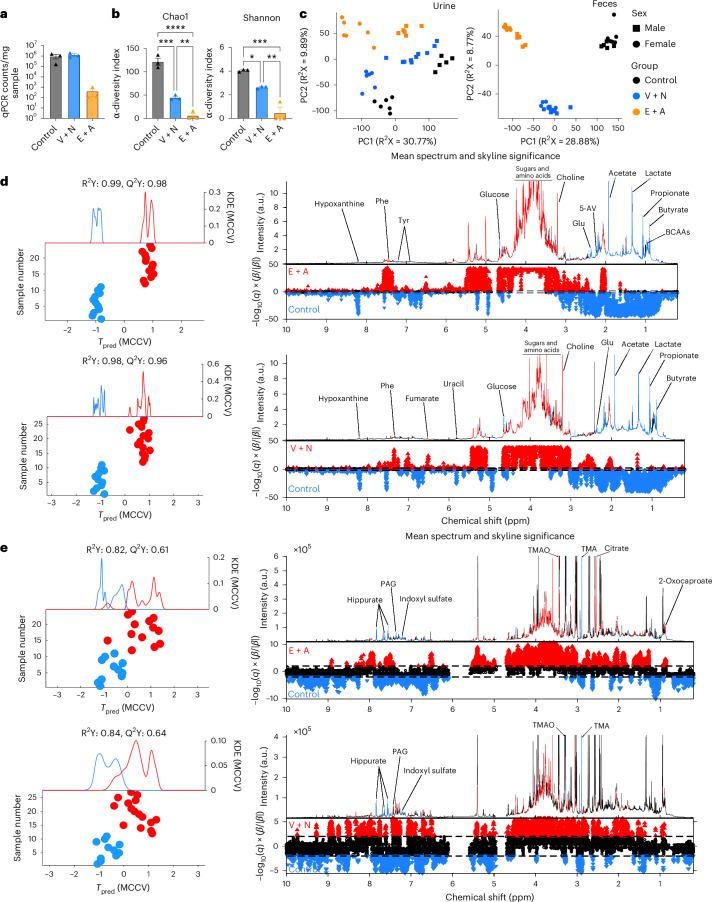
Effects of antibiotic regimens on gut bacterial abundance, diversity and host metabolic profiles. **a**, qPCR quantification of total bacterial 16S rRNA gene copies in control and antibiotics-treated mice. **b**, Fecal bacterial α-diversity measures from control and antibiotics-treated mice. In **a** and **b**, data are presented as mean ± standard error of mean (*n* = 3 mice per group). Statistical analysis was performed using one-way ANOVA with Tukey’s multiple comparisons test (*P < 0.05, **P < 0.01, ***P < 0.001, ****P < 0.0001). **c**, PCA score plots of ^1^H NMR spectral data from feces and urine following 1 week of treatment. PC1 and PC2 are the first and second principal components, and R^2^X indicates percentages of variation explained by each component. **d**,**e**, CA-PLS models, adjusted for sex, comparing metabolic profiles of feces (**d**) and urine (**e**) between controls and E + A- or V + N-treated mice. Plots show the predictive component (*T*_pred_) separating groups, with model predictive ability (Q^2^Y) evaluated by 100 rounds of Monte Carlo cross-validation (MCCV). Kernel density estimates (KDE) illustrate sample distribution along *T*_pred_. Q^2^Y indicates model predictive ability. The right panels display the mean NMR spectra (top), showing metabolic composition differences, while skyline significance plots highlight variable contributions to the models (red, antibiotic-treated samples; blue, controls). In **c**–**e**, sample sizes were as follows: control (male *n* = 5, female *n* = 6), V + N (male *n* = 10, female *n* = 6), E + A (male *n* = 6, female *n* = 7). 5-AV, 5-aminovalerate; a.u., arbitrary units; BCAAs, branched-chain amino acids; Glu, glutamate; PAG, phenylacetylglycine; Phe, phenylalanine; TMA, trimethylamine; TMAO, trimethylamine *N*-oxide; Tyr, tyrosine.

Quantitative polymerase chain reaction (qPCR) of total bacterial 16S rRNA gene copies in fecal samples showed a reduction, although not statistically significant, in bacterial load in the E + A group compared with the other groups (Fig. 2a). α-diversity, calculated from 16S rRNA gene-based sequencing data of three randomly selected fecal samples per group at the end of 1-week treatment, confirmed bacterial changes in the E + A and V + N groups (Fig. 2b). The E + A and V + N groups showed depletion of taxa dominant in control animals; however, V + N also showed enrichment of several families, including Enterobacteriaceae, Akkermansiaceae, Sutterellaceae and Tannerellaceae, suggesting a restructuring of the bacterial community rather than effective depletion (Supplementary Fig. 1). The ^1^H nuclear magnetic resonance (NMR) spectral data of urine and feces post-antibiotic treatment revealed clear sex- and antibiotic-dependent clustering in principal component analysis (PCA) score plots, with fecal profiles primarily driven by antibiotic treatment and urinary profiles by sex (Fig. 2c). To further assess antibiotic regimen impact on metabolic profiles, we conducted covariate-adjusted projection to latent structures (CA-PLS) models adjusting for sex^16^. Compared with controls, both antibiotic regimens induced a complete shift in metabolic profiles, highlighting the suppression of metabolic and microbial functions in the gut (Fig. 2d,e). In feces, both treatments led to increased choline and sugar levels and a marked reduction in bacterial fermentation products, including trimethylamine and short-chain fatty acids. Urinary concentrations of host–microbial co-metabolites, including hippurate, indoxyl sulfate, phenylacetylglycine, trimethylamine and trimethylamine *N*-oxide, were reduced.

While we did not directly compare AMVN with E + A or V + A, a previous study showed greater bacterial depletion with the E + A regimen than AMVN^3^. Antibiotics administered through drinking water may lead to variability in dosing due to differences in water intake between animals; however, it has been shown to achieve more effective bacterial depletion than once-daily OG with reduction of the overgrowth of opportunistic taxa such as *Escherichia*/*Shigella*^17^. Antifungal agents were not used as this was a short-term experiment; their addition may be considered depending on the experimental duration and conditions^18^.

Our data have led us to conclude that both E + A and V + N antibiotic combinations are well tolerated when administered via drinking water, eliminating the need for OG and thereby reducing handling stress and procedural risks. Considering both depletion efficacy and antibiotic-resistance concerns related to vancomycin^19^, E + A combination may represent a more suitable choice for gut bacterial depletion.

## Methods

### Experimental design and sample collection

All animal procedures were conducted under a Home Office UK-issued license (P9718F9C8) and in compliance with animal welfare regulations. Fabpl*Cre*;*Apc*^15lox/+^ mice, a model of colorectal cancer^15^, were bred under specific pathogen-free conditions at the Central Biomedical Services units of Imperial College London. Mice were maintained under standard environmental conditions (22 °C, 60–70% humidity, 12 h–12 h day–night cycle), with standard chow diet (Ssniff; product code 1534-00) and water provided ad libitum throughout the experiment.

#### Treatment with AMVN cocktail

Following 1 week of acclimatization, 7-week-old Fabpl*Cre*;*Apc*^15lox/+^ mice (male *n* = 17, female *n* = 20) were randomly assigned to AMVN (1 g/l ampicillin, 1 g/l metronidazole, 0.5 g/l vancomycin and 1 g/l neomycin in drinking water) (male *n* = 12 housed in 7 cages, female *n* = 15 in 6 cages) or control (male *n* = 5 housed in 2 cages, female *n* = 5 in 1 cage) groups. After 1 week, due to significant body weight loss, antibiotic administration was switched to OG of 100 µl AMVN once every 3 days. Mice were monitored for two additional weeks, recording body weight and food intake, with body weight measured weekly and food intake monitored per cage weekly.

#### Treatment with vancomycin–neomycin or enrofloxacin–ampicillin

Fabpl*Cre*;*Apc*^15lox/+^ mice aged 7 weeks old (male *n* = 21, female *n* = 19) were acclimatized for 1 week, then randomized based on body weight into control (male *n* = 5 in 2 cages, female *n* = 6 in 1 cage), vancomycin–neomycin (V + N: 0.5 g/l vancomycin, 1 g/l neomycin, male *n* = 10 in 3 cages; female *n* = 6 in 1 cage) or enrofloxacin–ampicillin (E + A: 0.575 g/l enrofloxacin, 1 g/l ampicillin, male *n* = 6 in 2 cages; female *n* = 7 in 2 cages) administered via drinking water for 1 week. The antibiotic-containing water was prepared fresh at the start of the treatment and remained unchanged during the treatment period. Enrofloxacin was less soluble in water compared to the other antibiotics, resulting in a cloudy solution. Body weight was measured daily; food and water intake were monitored per cage daily. Fecal and urinary samples were collected 1 day before antibiotic treatment and at the end of the 1-week course, then stored at −80 °C until processing.

### Sample preparation for ^1^H NMR spectroscopy

A 1.5 M potassium phosphate buffer (pH 7.4) was prepared using 100% D_2_O, 1 mg/ml 3-(trimethylsilyl)-[2,2,3,3-^2^H_4_] propionic acid sodium salt (TSP, for chemical shift calibration) and 0.13 mg/ml sodium azide (NaN_3_, bacteriostatic reagent). Urinary samples were thawed at room temperature and homogenized by vortexing for 5 s. A volume of 30 μl of urine samples was diluted with 33 μl of D_2_O, then mixed with 1.5 M potassium phosphate buffer at a 9:1 ratio (63 μl of diluted urine to 7 μl of buffer). A total of 60 μl of the final mixture was transferred into NMR tubes with an outer diameter of 1.7 mm. Approximately 30–50 mg of fecal samples were weighed, and extraction was performed using a 20× dilution of the 1.5 M potassium phosphate buffer at a 1:4 ratio (1 mg of sample to 4 μl of diluted buffer). Samples were then vigorously vortexed for 5 min and subjected to centrifugation at 20,000*g* for 20 min at 4 °C. A total of 60 μl of supernatant was transferred into 1.7 mm outer-diameter NMR tubes.

### ^1^H NMR spectral data acquisition and preprocessing

^1^H NMR spectroscopy was performed using a Bruker DRX 600 MHz spectrometer (Bruker DRX) utilizing a standard one-dimensional pulse sequence consisting of a recycle delay, 90° pulse, *t*_1_, 90° pulse, mixing time (*t*_m_), 90° pulse, and free induction decay acquisition. A recycle delay of 4 s and a mixing time of 100 ms were applied^20^. For spectral acquisition, 32 and 64 scans were collected for feces and urine, respectively. A line broadening factor of 0.3 Hz was applied before Fourier transformation. Spectral processing, including automated phasing, baseline correction and chemical shift calibration to the TSP peak (δ^1^H 0.0), was conducted using TopSpin (version 4.0). The processed spectra (δ^1^H 0–10 ppm) were imported into MATLAB (version R2018b, The MathWorks) with a resolution of 0.00035 ppm. Regions corresponding to TSP, water (δ^1^H 4.55–5.00 ppm) and urea (δ^1^H 5.535–6.075 ppm for urinary spectra) were excluded from analysis. To account for peak shifting due to pH variations, spectral alignment was performed^21^. Probabilistic quotient normalization was applied to correct for sample dilution differences^22^. Metabolite identification was achieved using statistical total correlation spectroscopy (STOCSY)^23^ and reference to the Chenomx NMR Suite (Chenomx).

### Statistical analysis of spectral data and metabolite quantification

Spectral data were mean-centered and scaled using the unit variance method before applying multivariate statistical analyses using MATLAB (version R2018b, The MathWorks). Initially, unsupervised PCA was used for dimensionality reduction and to visualize inherent differences between spectra. Subsequently, supervised CA-PLS^16^ analysis was applied, including sex as a covariate, to identify metabolites significantly altered between control and treatment groups. Robust cross-validation was achieved using 100 Monte Carlo rounds to measure mean cross-validated predictive component score (*T*_pred_) and variance for each sample. Model fit and predictive ability were assessed by R^2^X and Q^2^Y, respectively. Gaussian kernel density estimates (KDE) were used to visualize the distribution of *T*_pred_ scores across treatment groups. A total of 100 bootstrap resamplings within each of the 100 models was performed to calculate variance, mean coefficient and a *P* value for each ppm variable. These P values underwent Storey’s multiple test correction, and metabolites with a false discovery rate (*q*) ≤0.01 were deemed significant. For graphical summary, Manhattan plots were generated to show −log_10_(*q*) multiplied by the sign of each variable’s regression coefficient, with dashed lines indicating the significance threshold on the log_10_ scale.

### DNA extraction of feces and PacBio 16S ribosomal RNA gene sequencing

DNA extraction was performed using the DNeasy PowerSoil Pro QIAcube Kit (Qiagen), following the manufacturer’s protocol. Homogenization and cell lysis were carried out using the Bullet Blender Storm (Next Advance) at speed 8 for 3 min, replacing the PowerLyzer 24 Homogenizer recommended by the manufacturer. The extracted DNA was eluted in 10 mM Tris–Cl buffer (pH 8.5), and its concentration was measured using the Qubit dsDNA Broad Range Assay (Invitrogen), according to the manufacturer’s instructions. DNA samples were then stored at –20 °C until needed. Sequencing libraries were generated by amplifying the V1–V9 regions of the 16S rRNA gene using PacBio-specific primers: a forward primer (5′GCATC/barcode/AGRGTTYGATYMTGGCTCAG3′) and a reverse primer (5′GCATC/barcode/RGYTACCTTGTTACGACTT3′). DNA samples were normalized to a concentration of 2 ng/µl, and amplification was performed using the KAPA HiFi HS ReadyMix (500 reactions; Roche Diagnostics). Unique PacBio barcode sequences were attached to both forward and reverse primers to enable multiplexed sequencing. The barcoded primers were organized into PCR plates, and sequencing was carried out on a Sequel IIe system using one SMRT Cell 8M, yielding an average of 11,998 reads per sample.

### 16S ribosomal RNA gene sequencing data analysis

Raw sequencing fastq files were processed using the DADA2 package (Version 1.22.0) within the RStudio environment (Version 4.2.3). In brief, initial sample filtering involved removing the first 19 nucleotides corresponding to the primers. Reads with a quality score below 30 were trimmed, and those exceeding two expected errors or containing unique sequences were excluded. Default DADA2 parameters were applied for sequence dereplication, merging, denoising, chimera elimination and inference of sample sequences. Sequences were then examined as one pool (pseudo-pooled) using DADA2 to infer amplicon sequencing variants. Taxonomic classification of amplicon sequencing variants was conducted using the SILVA reference database (Version 138.1). α-diversity analysis was assessed in R (version 4.4.2) and RStudio (version 2024.12.1 + 563) using the phyloseq package to calculate Chao1 and Shannon diversity indices. Differences in α-diversity between experimental groups were assessed in GraphPad Prism 9 using the right statistical test based on data normality and corrected for multiple testing.

### qPCR

qPCR targeting the 16S rRNA gene was used to estimate total bacterial biomass in each sample, as previously performed^24^. Each reaction was carried out in a 20-μl volume containing 1× KAPA2G Fast HotStart ReadyMix (Roche) with ROX (Life Technologies), 1.8 μmol/l of the BactQUANT forward primer (5′-CCTACGGGAGGCAGCA-3′) and reverse primer (5′-GGACTACCGGGTATCTAATC-3′), 225 nmol/l of the probe ([6FAM] 5′-CAGCAGCCGCGGTA-3′ [MGBNFQ]), PCR-grade water (Roche) and 5 μl of DNA. Each PCR plate included a standard curve prepared from *Escherichia coli* DNA (Sigma-Aldrich) ranging from 3,500 to 350,000,000 copies per reaction in 10-fold serial dilutions, along with no-template controls. All samples, standards and controls were run in triplicate. Amplification and real-time fluorescence detection were performed on the Applied Biosystems StepOnePlus Real-Time PCR System (Applied Biosystems) using the following cycling program: 50 °C for 3 min, 95 °C for 10 min, followed by 40 cycles of 95 °C for 15 s and 60 °C for 1 min.

### Reporting summary

Further information on research design is available in the Nature Portfolio Reporting Summary linked to this article.

## Online content

Any methods, additional references, Nature Portfolio reporting summaries, source data, extended data, supplementary information, acknowledgements, peer review information; details of author contributions and competing interests; and statements of data and code availability are available at 10.1038/s41684-026-01728-3.

## Supplementary information

Supplementary Information Supplementary Fig. 1.

Reporting Summary

## Data Availability

The datasets used and/or analyzed during the current study are available from the corresponding author upon reasonable request.
