# Supplementary material for: Alternative antibiotic regimens improve palatability and welfare in mice for gut bacterial depletion
Source: Lab Anim (NY). 2026 May 11;55(7):271–4. doi: 10.1038/s41684-026-01728-3 (PMC13318628; doi:10.1038/s41684-026-01728-3)
Supplement: Supplementary file 1 — Supplementary Fig. 1. [file 41684_2026_1728_MOESM1_ESM.pdf]

lab animal

Supplementary information

https://doi.org/10.1038/s41684-026-01728-3

Alternative antibiotic regimens improve palatability and welfare in mice for gut bacterial depletion

In the format provided by the authors and unedited

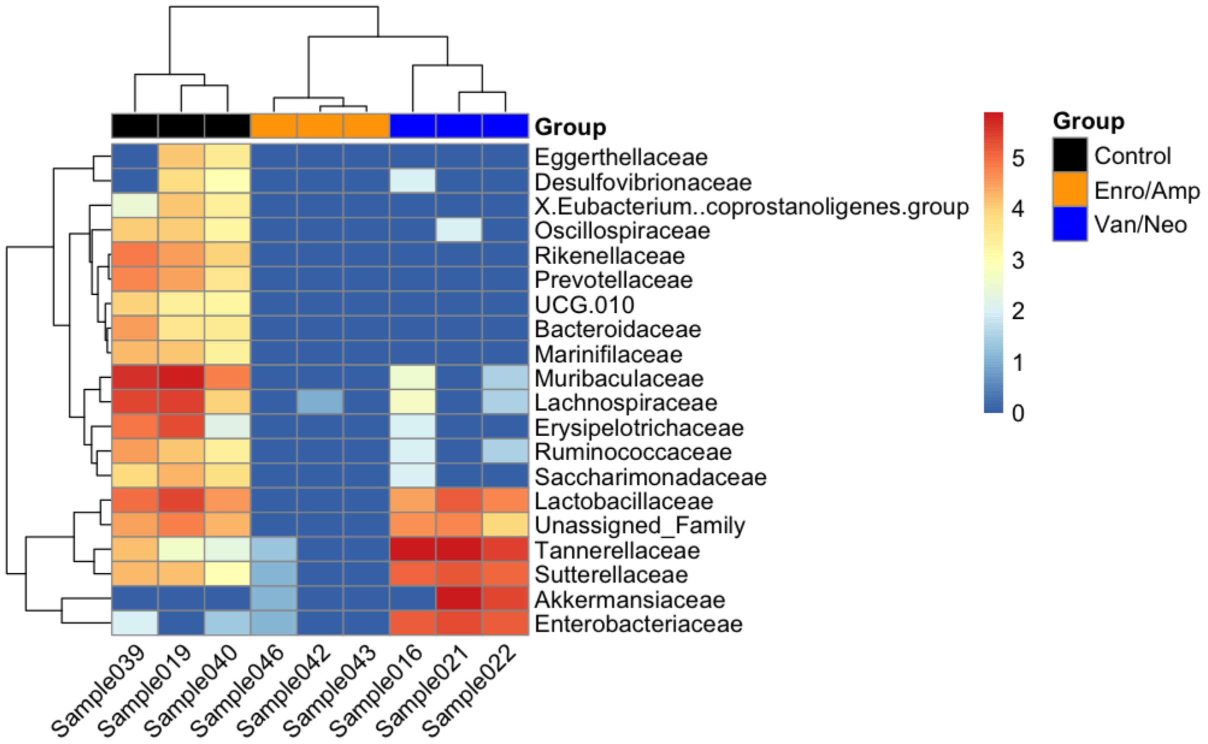

Figure S1: Absolute abundance of bacterial families. Heatmap showing the estimated absolute abundance of fecal bacterial families in control, enrofloxacin-ampicillin (E+A) and vancomycin-neomycin (V+N) treated mice. Absolute abundance was estimated by scaling relative 16S rRNA sequencing data with total bacterial load measured by qPCR and visualized as log10-transformed 16S rRNA gene copy numbers. Rows represent bacterial families and columns represent individual samples. Hierarchical clustering was applied to both taxa and samples.
