# Supplementary material for: Alternative antibiotic regimens improve palatability and welfare in mice for gut bacterial depletion
Source: Lab Anim (NY). 2026 May 11;55(7):271–4. doi: 10.1038/s41684-026-01728-3 (PMC13318628; doi:10.1038/s41684-026-01728-3)
Supplement: Supplementary file 2 — Reporting Summary [file 41684_2026_1728_MOESM2_ESM.pdf]

nature portfolio

Corresponding author(s): LABAN-BC02461

Last updated by author(s): Jul 25, 2025

Reporting Summary

Nature Portfolio wishes to improve the reproducibility of the work that we publish. This form provides structure for consistency and transparency in reporting. For further information on Nature Portfolio policies, see our Editorial Policies and the Editorial Policy Checklist.

Statistics

For all statistical analyses, confirm that the following items are present in the figure legend, table legend, main text, or Methods section.

n/a	Confirmed	
☐	☒	The exact sample size (n) for each experimental group/condition, given as a discrete number and unit of measurement
☐	☒	A statement on whether measurements were taken from distinct samples or whether the same sample was measured repeatedly
☐	☒	The statistical test(s) used AND whether they are one- or two-sided<br>*Only common tests should be described solely by name; describe more complex techniques in the Methods section.*
☐	☒	A description of all covariates tested
☐	☒	A description of any assumptions or corrections, such as tests of normality and adjustment for multiple comparisons
☐	☒	A full description of the statistical parameters including central tendency (e.g. means) or other basic estimates (e.g. regression coefficient) AND variation (e.g. standard deviation) or associated estimates of uncertainty (e.g. confidence intervals)
☒	☐	For null hypothesis testing, the test statistic (e.g. F, t, r) with confidence intervals, effect sizes, degrees of freedom and P value noted<br>*Give P values as exact values whenever suitable.*
☒	☐	For Bayesian analysis, information on the choice of priors and Markov chain Monte Carlo settings
☒	☐	For hierarchical and complex designs, identification of the appropriate level for tests and full reporting of outcomes
☒	☐	Estimates of effect sizes (e.g. Cohen's d, Pearson's r), indicating how they were calculated

Our web collection on statistics for biologists contains articles on many of the points above.

Software and code

Policy information about availability of computer code

Data collection	NMR spectra were collected using Bruker TopSpin (version 4.0). 16S rRNA gene sequencing was performed using the PacBioo Sequel IIe.
Data analysis	Microbiome sequencing data were processed using the DADA2 package (version 1.22.0) in R (version 4.2.3) within RStudio. Amplicon sequence variant inference, chimera removal, and taxonomic classification (SILVA v138.1) were performed using default DADA2 parameters. Diversity metrics were calculated using the phyloseq package (R version 4.4.2; RStudio version 2024.12.1+563).<br>Multivariate modeling (CA-PLS) was performed using custom MATLAB code (version 2018b), developed at Imperial College London, as cited in the Methods. Univariate statistical analyses were performed using GraphPad Prism (version 9).

For manuscripts utilizing custom algorithms or software that are central to the research but not yet described in published literature, software must be made available to editors and reviewers. We strongly encourage code deposition in a community repository (e.g. GitHub). See the Nature Portfolio guidelines for submitting code & software for further information.

Data

Policy information about availability of data

All manuscripts must include a data availability statement. This statement should provide the following information, where applicable:

- Accession codes, unique identifiers, or web links for publicly available datasets
- A description of any restrictions on data availability
- For clinical datasets or third party data, please ensure that the statement adheres to our policy

The datasets used and/or analyzed during the current study are available from the corresponding author upon reasonable request.

Research involving human participants, their data, or biological material

Policy information about studies with human participants or human data. See also policy information about sex, gender (identity/presentation), and sexual orientation and race, ethnicity and racism.

Reporting on sex and gender	N/A
Reporting on race, ethnicity, or other socially relevant groupings	N/A
Population characteristics	N/A
Recruitment	N/A
Ethics oversight	N/A

Note that full information on the approval of the study protocol must also be provided in the manuscript.

Field-specific reporting

Please select the one below that is the best fit for your research. If you are not sure, read the appropriate sections before making your selection.

☒ Life sciences ☐ Behavioural & social sciences ☐ Ecological, evolutionary & environmental sciences

For a reference copy of the document with all sections, see nature.com/documents/nr-reporting-summary-flat.pdf

Life sciences study design

All studies must disclose on these points even when the disclosure is negative.

Sample size	No statistical power calculation was performed. Sample sizes were based on prior studies using metabolic and microbial profiling endpoints.
Data exclusions	No data were excluded for metabolomics analysis. For 16S rRNA gene sequencing analysis, a subset of mice in each group were analysed. This is because we have used metabolomics to assess the gut microbial function which is a more important compared to compositional changes post antibiotics.
Replication	No technical replicates were performed.
Randomization	Mice were randomized based on bodyweight prior to allocation to antibiotic groups. Samples were randomized prior to analyses.
Blinding	Blinding was not implemented, as antibiotic treatments involved distinct handling that made group allocation apparent. During sample 1H NMR and 16S rRNA sample processing, each sample was assigned an unique ID without revealing the group identity and all samples were then randomized prior to data acquisition. Both unsupervised PCA (no requirement of group identity) and supervised modeling method (group identity required) were performed in the current study.

Reporting for specific materials, systems and methods

We require information from authors about some types of materials, experimental systems and methods used in many studies. Here, indicate whether each material, system or method listed is relevant to your study. If you are not sure if a list item applies to your research, read the appropriate section before selecting a response.

Materials & experimental systems

n/a	Involved in the study
☒	☐ Antibodies
☒	☐ Eukaryotic cell lines
☒	☐ Palaeontology and archaeology
☐	☒ Animals and other organisms
☒	☐ Clinical data
☒	☐ Dual use research of concern
☒	☐ Plants

Methods

n/a	Involved in the study
☒	☐ ChIP-seq
☒	☐ Flow cytometry
☒	☐ MRI-based neuroimaging

Animals and other research organisms

Policy information about studies involving animals; ARRIVE guidelines recommended for reporting animal research, and Sex and Gender in Research

Laboratory animals: Mouse strain FabplCre;Apc15lox/+ aged 7 weeks old.

Wild animals: N/A

Reporting on sex: Both male and female mice were included in the study. Sex distribution and group sizes are reported in the figure legends. Sex-based analyses were not performed separately; however, multivariate modeling (CA-PLS) was used to correct for the impact of sex and isolate the effect of antibiotic treatment.

Field-collected samples: N/A

Ethics oversight: All animal procedures were conducted under a Home Office UK issued license (P9718F9C8) and in compliance with animal welfare regulations.

Note that full information on the approval of the study protocol must also be provided in the manuscript.

Plants

Seed stocks: N/A

Novel plant genotypes: N/A

Authentication: N/A
